# BRAZILIAN CONSENSUS FOR MULTIMODAL TREATMENT OF COLORECTAL LIVER METASTASES. MODULE 3: CONTROVERSIES AND UNRESECTABLE METASTASES

**DOI:** 10.1590/0102-6720201600030011

**Published:** 2016

**Authors:** Orlando Jorge Martins TORRES, Márcio Carmona MARQUES, Fabio Nasser SANTOS, Igor Correia de FARIAS, Anelisa Kruschewsky COUTINHO, Cássio Virgílio Cavalcante de OLIVEIRA, Antonio Nocchi KALIL, Celso Abdon Lopes de MELLO, Jaime Arthur Pirola KRUGER, Gustavo dos Santos FERNANDES, Claudemiro QUIREZE JR, André M. MURAD, Milton José de BARROS E SILVA, Charles Edouard ZURSTRASSEN, Helano Carioca FREITAS, Marcelo Rocha CRUZ, Rui WESCHENFELDER, Marcelo Moura LINHARES, Leonaldson dos Santos CASTRO, Charles VOLLMER, Elijah DIXON, Héber Salvador de Castro RIBEIRO, Felipe José Fernandez COIMBRA

**Affiliations:** 1 Brazilian Chapter of the International Hepato-Pancreato Biliary Association (BC-IHPBA);; 2 Brazilian Society of Surgical Oncology (BSSO);; 3 Brazilian Society of Clinical Oncology (BSCO);; 4 Brazilian College of Digestive Surgery (BCDS);; 5 Brazilian College of Surgeons (BCS),; 6 Americas Hepato-Pancreato-Biliary Association - AHPBA;; * Member of the Brazilian Society of Interventional Radiology, Brazil

**Keywords:** Colorectal neoplasms, Neoplasm metastasis, Drug therapy

## Abstract

In the last module of this consensus, controversial topics were discussed. Management of the disease after progression during first line chemotherapy was the first discussion. Next, the benefits of liver resection in the presence of extra-hepatic disease were debated, as soon as, the best sequence of treatment. Conversion chemotherapy in the presence of unresectable liver disease was also discussed in this module. Lastly, the approach to the unresectable disease was also discussed, focusing in the best chemotherapy regimens and hole of chemo-embolization.

## RESUMO

Neste último módulo do consenso, abordou-se alguns temas controversos. O primeiro tópico discutido foi o manejo da doença após progressão na primeira linha de quimioterapia, com foco em se ainda haveria indicação cirúrgica neste cenário. A seguir, o painel debruçou-se sobre as situações de ressecção da doença hepática na presença de doença extra-hepática, assim como, qual a melhor sequência de tratamento. O tratamento de conversão para doença inicialmente irressecável também foi abordado neste módulo, incluindo as importantes definições de quando se pode esperar que a doença se torne ressecável e quais esquemas terapêuticos seriam mais efetivos à luz dos conhecimentos atuais sobre a biologia tumoral e taxas de resposta objetiva. Por último, o tratamento da doença não passível de ressecção foi discutida, focando-se nos melhores esquemas a serem empregados e seu sequenciamento, bem como o papel da quimioembolização no manejo destes pacientes.

## INTRODUCTION

Closing the sequence of papers on the First Brazilian Consensus of Colorectal Liver Metastases, in this one controversial themes on this multimodality treatment are discussed. The first section refers to the management of resectable liver disease that progressed while on first-line chemotherapy; then the approach to patients with extrahepatic disease is discussed. The next topic was conversion systemic treatment in order to achieve resectability, followed by some discussion regarding the surgical treatment and the strategies adopted to avoid the occurrence of postoperative liver failure. Finally, an analysis of palliative systemic treatment was performed, with focus on the different regimens and their sequence, and also about different locoregional modalities in this setting. 

## METHOD

The same methodology of this consensus previous discussions was adopted, which included a literature review and the a discussion of topics by a Experts Committee prior to the consensus meeting, in which their conclusions were presented, followed by a debate and voting by the event attendees. Consensus was reached when over 75% of agreement was obtained after voting.

### TOPIC 8: Treatment choices in progression after first line chemotherapy in resectable disease

The role of chemotherapy in the treatment of patients with resectable colorectal liver metastases has gained importance after the gains in progression-free survival (PFS) observed in a randomized study^1^. However, the most appropriate time, whether neo- or adjuvant, remains a controversial issue in the literature^2^. In the neoadjuvant setting, evaluation of in vivo response allows a better selection of candidates for surgery, since the sensitivity of the tumor to chemotherapy has been shown to be an important prognostic factor^3,4,5,6,7,8,9,10 11^.

However, the risk of progression, making a patient ineligible for surgery, is seen as an eventual disadvantage of neoadjuvant treatment. This risk, however, has been shown to be less than 10% with the use of more modern regimens^3^. In general, these patients make up a very heterogeneous population with regard to the progression of sites and other clinical and prognostic factors, allowing different therapeutic approaches ranging from exclusive palliative chemotherapy to more complex treatments with a multidisciplinary approach involving surgery, imaging and interventionist radiology. 

The combination of these factors has resulted in scant literature on the approach to these patients, composed mainly of uni-institutional retrospective series and few banks of prospective data. These publications involve a small number of patients, which despite the progression of chemotherapy, underwent resection for liver disease^3^ ^,^ ^11^ ^,^ ^12^ ^,^ ^13^ ^,^ ^14^. Moreover, they feature a heterogeneous population, selected through varied inclusion/exclusion regarding the number and chemotherapy regimen used, the presence of extrahepatic disease or other prognostic factors. The impact of this diversity may have influenced the different results in survival found in the published studies. Adam et al., Kornprat et al. and Haas et al. observed survival at five years (OS5y) less than 10%, and thus similar to that expected in series of patients treated with chemotherapy alone^11^ ^,^ ^12^ ^,^ ^15^. However, Neumann et al., Gallagher et al. and Vigano et al. found OS5y of 36%, 61% and 35%, respectively^3^ ^,^ ^13^ ^,^ ^14^. These results suggest the existence of a population with potential benefit to surgery, however, with great importance to the adequate selection of these patients in the context of an approach in multidisciplinary meeting. Factors related to clinical presentation at time of progression, co-morbidities, length of surgery, imminent risks facing progression and other prognostic factors should be considered in the therapeutic approach. In this sense, robust data does not exist in the literature to aid in this decision. 

LiverMetSurvey data involving 175 patients undergoing hepatectomy after progression of disease, stress the importance of lesion size (≥50 mm), number of lesions (>3) and CEA≥200 as adverse prognostic factors in this population^3^. While not a predictive factor of benefit to surgery, the recognition of prognostic factors, for example, should be considered in the management of these patients. 

On the other hand, the benefit of second-line chemotherapy is based on robust data with several phase III studies. These studies have shown median survival of approximately 10 months and few patients alive at five years16,17,18. However, these results should be interpreted with some limitation to the population in question, since these studies involve non-selected patients, being mostly represented by non-candidates to surgery. Some series, however, also suggest that patients undergoing second-line chemotherapy can still benefit from a surgical approach. In retrospective data from M.D. Anderson Cancer Center involving 60 patients, Brouquet et al. observed recurrence-free survival (RFS) of 11% and OS5y of 22%19. Thus, it is considered that patients with resectable liver disease after progression and chemotherapy, in both first-line and second-line, should have their therapeutic approach discussed in multidisciplinary meeting.

### RECOMMENDATIONS

• All cases of disease progression after first line chemotherapy in the presence of resectable liver disease should be discussed in multidisciplinary meeting in the presence of a skilled surgeon, oncologist, interventional radiologist and a radiologist. 

Agreement: 97%

• In this scenario, resection of liver metastases is considered after multidisciplinary discussion in selected patients with good prognostic factors and favorable clinical and surgical conditions. Despite the poor prognosis, patients with progression after chemotherapy can still benefit from hepatectomy, reaching higher survival rates than those observed with chemotherapy alone. 

Agreement: 87%

• Chemotherapy is recommended for patients with other risk factors or unfavorable clinical and/or surgical conditions. 

Agreement: 100%

• Discussions in multidisciplinary board are still recommended for the evaluation of the surgical approach in accordance with second-line treatment evolution. 

Agreement: 93%

### TOPIC 9: Management in the presence of extrahepatic disease

In order to formulate a guideline for the treatment of patients with colorectal cancer with hepatic and extrahepatic metastases, the panel of experts carried out an extensive literature review (see description in the editorial referring to this consensus) associated with critical analysis of the members of the consensus in order to answer important practical issues in the management of metastatic colorectal cancer, namely:

What is the best method for the definition of extrahepatic disease?

What is the impact of the various sites on survival?

Is there a role for hepatic resection in patients with resectable extrahepatic disease?

What is the surgical treatment sequence with regard to extrahepatic sites and liver metastases?

In an analysis of the literature, the panel of experts identified computed tomography (CT) as the preferred method for the diagnosis of extrahepatic disease^20^ ^,^ ^21^ ^,^ ^22^ ^,^ ^23^ ^,^ ^24^. It is the method of choice for staging and follow-up of patients with colorectal cancer, as imaging methods are widespread in our environment, familiar to oncologists, radiologists and surgeons, with good cost/benefit. Thus, the use of CT is recommended as the initial method in the diagnosis of extrahepatic metastases.

The use of PET-CT has a complementary role in the evaluation of patients with liver metastases and in other sites. It often detects other sites beyond those suspected by CT (up to 48% of cases), resulting in increased clinical-radiological staging. In severe circumstances of the patient with metastases in multiple sites, the finding of new lesions often implies change in therapeutic strategies (20-50% of cases) and prevents unnecessary operations^20^ ^,^ ^21^ ^,2220,^ ^21^ ^,^ ^22^. Despite having limited access in many centers in our country, PET-CT is recognized to be beneficial in selecting patients for hepatectomy and patients with metastases in liver and other sites. Additionally, PET-CT is recommended in initial staging, before any systemic treatment when the identification of hepatic and extrahepatic lesions and staging with CT as base. This course of treatment would avoid the negative effects of chemotherapy over the sensitivity of PET-CT and serve as a guide to future local treatments for metastases. From the above, PET-CT is recognized and should be performed on the patient with liver metastases and extrahepatic, whenever available, at the center that treats the patient.

The use of invasive diagnostic methods - biopsies - were not assessed in any paper in the literature. After multidisciplinary discussion during the course of consensus, it was understood that evaluation by biopsy should be indicated in cases where non-invasive methods (CT and PET-CT) are not able to define the presence of metastasis/recurrence and, above all, if the biopsy result implies therapeutic change.

The occurrence of metastases in other concomitant organs to liver lesions implies drastic reduction in the survival rate of these patients and frequently puts them in a palliative care situation. Nevertheless, there are situations where surgical treatment involves a gain of survival. In this fact lies the importance of multidisciplinary assessment from the beginning of treatment to identify potential candidates for surgical treatment^25^ ^,^ ^26^ ^,^ ^27^. Table 1 shows the impact of different metastatic sites in patients with secondary liver lesions.

**TABLE 1:** Patients undergoing surgical treatment of hepatic and extrahepatic metastases: incidence of extra-hepatic lesions and survival after treatment

Site	Incidence (relative to the total of patients with extrahepatic disease submitted to surgery)	Mean survival
Lung	27-51%	39-98 months
Peritoneum	12-15%	18-32 months
Lymph nodes (hepatic hilum, celiac trunk, aortocaval)	6.7-32%	13-48 months
Others (ovary, adrenal, bone)	2-16%	16-82 months
Multiple sites	8-10.5%	15-18 months

This consensus meeting considered the indicated operative treatment in patients selected based on two criteria^25^ ^,^ ^26^ ^,^ ^27^ ^,^ ^28^ ^,^ ^29^. First, favorable tumor biological behavior to chemotherapy treatment. Chemotherapy regimens will be discussed in another section of this consensus, but it is to say that patients with multiple colorectal cancer metastases are carriers of systemic disease, and as redundancies are part, require systemic control of cancer. Chemotherapeutic treatment allows a temporal analysis of disease progression as well as evaluates its sensitivity to medications prescribed. The second feature that guides surgical treatment is the direct responsibility of the surgeon: the ability of complete resection of all affected sites. Important to note is that surgical treatment results in improved survival when metastases affect the liver and one other site; in cases of cancer with secondary implants at multiple sites (liver and two or more other organs) survival is usually reduced and there is no room for salvage surgery^25^. 

Treatment should occur when there is response to systemic treatment and the lesions are resectable. When there is no intention of surgical treatment, no indication to indefinitely extend chemotherapy treatment, or in other words, once the favorable biological response is observed and there is a possibility of complete resection of all sites, resection is to be performed. 

As for the surgical treatment sequence, it should be started with the more complex surgery that will most likely prevent complete resection of the target lesions. Usually the liver is the site of the largest number of tumors and, in cases of multiple metastases, requires association of complex interventions (staged hepatectomy, portal occlusion, radioablation). In this scenario, the liver will generally be approached first, followed by the other sites (lung, peritoneum, etc.). Less frequently is the patient approach with extrahepatic disease occurring in reverse, for example, in cases of complex locoregional recurrence or multiple lung lesions in patients with uninodular liver disease. In these exceptional cases, the liver can be approached after the extrahepatic lesion.

It is common to consider simultaneous surgical resection in patients with resectable lesions in the liver and other organs. The indication should be selective, taking into consideration the biological behavior described above, the possibility of complete resection in all sites and surgical size of aggregate operations. 

According to literature data, hepatectomy and concomitant resection of extrahepatic disease is indicated in the following situations^25^ ^,^ ^26^ ^,^ ^27^ ^,^ ^28^ ^,^ ^29^ ^,^ ^30^ ^,^ ^31^ ^,^ ^32^ ^,^ ^33^ ^,^ ^34^ ^,^ ^35^ ^,^ ^36^ ^,^ ^37^ ^,^ ^38^ ^,^ ^39^ ^,^ ^40^ ^,^ ^41^ ^,^ ^42^ ^,^ ^43^ ^,^ ^44^ ^,^ ^45^ ^,^ ^46^ ^,^ ^47^ ^,^ ^48^ ^,^ ^49^ ^,^ ^50^ ^,^ ^51^.

1. Lymph nodes affected in the hepatic hilum: those patients with hepatic hilar lymph node recurrence and presenting favorable response to systemic treatment, a lymphadenectomy of the hepatic hilum will result in survival of 25% at five years. Palliative-only treatment offers worse survival (null). Lymph node recurrence in distant lymphatic chains, as celiac trunk and aortocaval, do not benefit from resection.
2. Peritoneal carcinomatosis: also involves selective indication and should take into consideration the number of hepatic lesions as well as the peritoneal carcinomatosis index (PCI). Patients who benefit from concomitant treatment are those with limited hepatic disease (<3 modules) and restricted peritoneal disease (PCI< 12).
3. Local recurrence (anastomosis recurrence, insufficient lymphadenectomy to primary tumor): follows the same recommendation of colorectal and synchronous hepatic resections. In this scenario, when there is local recurrence, this should be addressed at first resection, since rates of unresectability of up to 50% have been reported.
4. Lung: despite case reports in the literature, in general, the elevated size of the surgery hinders synchronic resection to occur safely.
5. Infrequent sites (adrenal, ovary): analyze course of treatment case-by-case in the environment of multidisciplinary discussion. Literature data is very scarce to offer scientific support to therapeutic decisions.

### RECOMMENDATIONS

• Best extrahepatic disease detection method is the Computed Tomography with IV contrast. Consensus recommends the use of PET-CT in this scenario when available.

Agreement: 88%

• Indicate resection in cases of favorable biological behavior, favorable risk factors with response to chemotherapy and favorable site (lung, peritoneum, lymph nodes, hepatic hilum, ovary, local recurrence).

Agreement: 85%

• Avoid hepatectomy if it is not possible to achieve complete resection of all sites.

Agreement: 82%

• Therapeutic sequence: Address the first site of the most therapeutic complexity, usually the liver (which limits complete resection).

Agreement: 88%

• Simultaneous approach in selected cases: liver and peritoneum (limited number of nodes and PCI); local recurrence and liver; ovary and liver, remembering that complex procedures must not be combined.

Agreement: 84%

### TOPIC 10: Conversion therapy in unresectable disease

Most patients with colorectal cancer that develop liver metastases are not amenable to resection in the diagnosis of metastatic disease (about 80-90% of cases). In this scenario, patients should be evaluated as to the possibility of conversion therapy or, if not possible, sent for treatment strategies in a palliative setting. The factors that associate with conversion capacity can be classified into clinical, biological and anatomical^52^.

In clinical evaluation of these patients, the biological age, comorbidities, nutritional status, performance status, ability to tolerate treatment (systemic and surgical) and social support should be considered^53^. What should be taken into account in this analysis and presentation of the case: whether the disease is restricted to the liver or associated with other sites of extrahepatic disease that could oncologically benefit with surgical treatment. Evaluate the biology or behavior of the disease by the result of systemic treatment response and control of metastatic disease and in the anatomical aspect, check the possibility of obtaining R0 resection in case of response to treatment, following the precepts of resectability previously described. The ultimate goals are controlled systemic disease and R0 resection^52^ ^,^ ^54^.

This multidisciplinary approach is aimed to adequately evaluate each patient so that the best strategy and treatment regimen can be defined. Ideally, radiologists and clinical oncologists should participate in this surgical decision (oncological, digestive, general or hepatobiliopancreatic) with experience in liver surgery^52^.

Regarding the choice of systemic chemotherapy protocol, the evidence points to a correlation between response rate and resection of liver metastases and this correlation is greater when calculated from studies of patients with hepatic-only goal, compared with studies of a general metastatic population. Despite the response rate outcome being used as a guide in choosing the therapeutic regimen, it is known that this is not the ideal outcome for correlating reliably with stronger outcomes, such as disease-free survival after resection. The definition of the therapeutic regimen goes further by mutational analysis of RAS and issues related to the patient, such as ability to tolerate the proposed regimen or any comorbidity that limits the use of any of the chemotherapeutic agents^54^. 

Taking into account the response rate data and tolerance to treatment, systemic treatment options are: 1) Patients with wild-type RAS: FOLFOX with panitumumab or cetuximab, FOLFIRI with panitumumab or cetuximab, FOLFOXIRI with or without bevacizumab, FOLFOX, XELOX or FOLFIRI with or without bevacizumab^55^ ^,^ ^56^ ^,^ ^57^ ^,^ ^58^ ^,^ ^59^ ^,^ ^60^. 2) Patients with mutated RAS: FOLFOXIRI with or without bevacizumab, FOLFOX or XELOX with or without bevacizumab, FOLFIRI with or without bevacizumab^16^ ^,^ ^59^ ^,^ ^60^. In patients with wild-type RAS, there is not a comparison between FOLFOXIRI with or without monoclonal antibody versus FOLFIRI or FOLFOX with anti-EGFR. However, based on the toxicity profile, there is a preference for the use of a less intense chemotherapy regimen associated with anti-EGFR. In patients with RAS mutation with clinical conditions to tolerate a more intense treatment, the initial preference would be for FOLFOXIRI with or without bevacizumab. Preferring FOLFIRI as conversion chemotherapy in the case of prior adjuvant therapy with FOLFOX ending less than 12 months and/or is associated with significant neuropathy.

Some observations as to conversion treatment: there is no data to support the use of routine monoclonal antibodies after resection; a paucity of evidence as to the benefit of irinotecan in postoperative setting with no residual disease; the role of systemic treatment change, in the absence of pathological response in post-resection surgical specimen, has yet to be established^61^ ^,^ ^62^ ^,^ ^63^ ^,^ ^64^.

During treatment, evaluation of response should be carried out through a multidisciplinary approach every 2 or 3 months with laboratory tests (including hematological tests, liver function, CEA tumor markers and CA19.9) and restaging by imaging (three-phase multidetector computed tomography and/or nuclear magnetic resonance, if possible with diffusion and liver-specific contrast), always compared to previous exams^65^. In the evaluation of response, use the criteria of RECIST 1.1^65^ ^,^ ^66^. The use of PET-CT is not supported for routine use in response evaluation, safeguarding its use for special situations. 

The time to program the resection should be decided together and generally indicates surgery, thereby the surgical team judges resectable lesions, respecting the established criteria of resectability and remnant liver. Delaying surgery when the lesions are already eligible for resection can lead to problems such as increased postoperative morbidity and missing metastases. The surgeries that are often necessary are: two-stage hepatectomy, with or without portal vein embolization or ligation of the portal vein. Radiofrequency can still be performed in association with surgery in livers with multiple lesions in which the future residual liver would not be enough, in lesions of up to 3 cm, lying at least 1 cm from the biliary tract. In general, surgery is programed 4-6 weeks from the last chemotherapy cycle and last 6-8 weeks from the last application of bevacizumab, if such has been employed^54^ ^,^ ^67^ ^,^ ^68^ ^,^ ^69^.

In the surgical evaluation of these patients eligible for resection after conversion therapy, a crucial point to be defined is the estimate of future remnant liver function. This data obviously depends on liver residual mass, but also other factors such as personal history of plurimetabolic syndrome and hepatopathy, as well as the number of cycles of chemotherapy to which the patient was exposed to before surgery^70^. Although some authors have related therapeutic regimens with specific lesions to non-tumor liver parenchyma and morbidities characteristics, which is consolidated in recent studies that show these findings correlate more strongly to the number of cycles than to the treatment regimen itself, more than 6-8 treatment cycles can significantly increase the risk of postoperative hepatic failure^71^ ^,^ ^72^. In these cases, the consensus advocates liberality in the use of techniques to increase the volume of future remnant liver, working with a minimum percentage of 30% of the total liver volume, as discussed in the specific session^73^. Additionally, during surgery, a meticulous surgical technique aimed at preserving the maximum of parenchyma possible and avoid the need for blood transfusions is advised, as these data are also associated with higher incidence of postoperative complications, among them liver failure. Other warning data in this scenario are the findings of intense steatosis in imaging tests, splenomegaly and other stigmata of portal hypertension and inadequate hypertrophy of the hepatic parenchyma after portal embolization in cases where it becomes necessary, reinforcing the need of the team's multidisciplinary expertise in the care of these patients.

### RECOMMENDATIONS

• Patients with inoperable disease confined to the liver (or resectable extrahepatic disease, with potential oncological benefit) should be candidates for conversion therapy and perspective candidates of R0 surgery in case of response. Should tolerate systemic treatment and the proposed surgical risk.

Agreement: 91%

• Chemotherapy regimens should be chosen for higher response rate (RR) shown because the correlation between RR and resection:

o Wild-type KRAS and NRAS: FOLFOX or FOLFIRI with panitumumab or cetuximab or FOLFOXIRI with or without bevacizumab or FOLFOX, XELOX, FOLFIRI with or without bevacizumab;

o Mutated KRAS or NRAS: FOLFOXIRI with or without bevacizumab, FOLFOX, XELOX or FOLFIRI with or without bevacizumab.

Agreement: 88%

• Evaluation of response should be performed every 2-3 months with serum markers (CEA, CA 19-9) and imaging exams (CT or MRI - RECIST) and surgery should be performed the moment an R0 resection is deemed possible by the surgical team 

Agreement: 95%

• There is no methodology with optimal accuracy for measuring the impact of chemotherapy on liver function. Use clinical, laboratory and radiographic data and liver biopsy in selected cases. Whenever possible, carry out the lowest number of cycles of chemotherapy, using liver hypertrophy techniques, measurement of future remnant liver, techniques aimed at saving parenchyma and rely on the surgical team with experience in oncological liver surgery.

Agreement: 97%

### TOPIC 11: Palliative treatment

#### Definitions and objectives

Treatment is defined as palliative when the disease is not amenable to resection even after complete conversion chemotherapy^74^ ^,^ ^75^.

In these cases, the main objectives are to increase overall survival and/or progression-free survival, with control of symptoms secondary to cancer and minimize the side effects of therapy.

#### First-line treatment

The treatment of incurable metastatic colorectal cancer is a continuum of sequential lines. It is important to identify a priori the criteria that impact the choice of treatment^76^. Recommend to evaluate:

a) status of KRAS and NRAS mutations. BRAF evaluation is optional.
b) clinical variables: volume of disease, presence of symptoms, age, performance status, comorbidities and the patient 's desire.

#### First-line chemotherapy options are:

a) FOLFOX, CAPOX or FOLFIRI regimens are equivalent^16^ ^,^ ^77^.
b) Monotherapy with capecitabine or fluorouracil is a valid option for frail patients.
c) FOLFOXIRI is valid option, but with greater toxicity^59^ ^,^ ^78^.
d) Infusional fluorouracil is preferred in relation to regimens in bolus^79^.

After obtaining the best response, valid options are: maintain the same treatment, maintain fluoropyrimidine (with or without monoclonal antibody) or chemotherapy-free interval. This decision is based on the set of clinical variables (disease volume, presence of symptoms, age, performance status, comorbidities, response to treatment and patient's desire)^80^ ^,^ ^81^ ^,^ ^82^.

Patients who are exposed to all available drugs have longer survival and patients receiving more early-line drugs are more likely to be exposed to all drugs^83^ ^,^ ^84^ ^,^ ^85^.

#### Options of first-line monoclonal antibodies:

a) Mutated KRAS or NRAS: do not use cetuximab or panitumumab^57^. Bevacizumab can be combined with FOLFOX, CAPOX, FOLFIRI or FOLFOXIRI^78^ ^,^ ^86^ ^,^ ^87^.

b) Wild-type KRAS and NRAS: bevacizumab, cetuximab or panitumumab can be combined with chemotherapy protocols. There is no definitive evidence on which combination is superior. Do not combine cetuximab or panitumumab to chemotherapy containing capecitabine. Bevacizumab can be combined with FOLFOX, CAPOX, FOLFIRI or FOLFOXIRI. Cetuximab and panitumumab can be combined with FOLFOX or FOLFIRI^55^ ^,^ ^57^ ^,^ ^76^ ^,^ ^77^ ^,^ ^86^ ^,^ ^87^ ^,^ ^88^ ^,^ ^89^.

Monoclonal antibodies should not be used in combination with each other, because the association is deleterious^90^ ^,^ ^91^.

#### Treatment in subsequent lines

Chemotherapy options in subsequent lines:

a) If first-line is based on oxaliplatin, use the regimen based on irinotecan or vice versa^16^ ^,^ ^83^.
b) If first-line is monotherapy with capecitabine or fluorouracil, consider oxaliplatin and sequential irinotecan (in any order). Oxaliplatin should not be used in monotherapy. Irinotecan can be used in monotherapy^84^ ^,^ ^85^.
c) After progression to fluoropyrimidine, capecitabine in monotherapy is not recommended.
d) After FOLFOXIRI, there is no standard chemotherapy regimen defined.

#### Monoclonal antibodies options and drug target in subsequent lines:

a) KRAS or NRAS mutated: do not use cetuximab or panitumumab. Bevacizumab can be used with FOLFOX, XELOX, FOLFIRI or irinotecan. Aflibercept can be used with FOLFIRI^92^ ^,^ ^93^ ^,^ ^94^.
b) Wild-type KRAS or NRAS: cetuximab or panitumumab is only indicated if there was no progression of these drugs in previous line. After progression to cetuximab, panitumumab is not indicated and vice versa. Cetuximab and panitumumab can be combined with FOLFOX, FOLFIRI or irinotecan. Cetuximab and panitumumab can be used as monotherapy and are equivalent between themselves. Bevacizumab can be used with FOLFOX, XELOX, FOLFIRI or irinotecan. Aflibercept can be used with FOLFIRI^58,92,95.96^.
c) Bevacizumab and aflibercept should not be used in monotherapy.
d) After progression to bevacizumab in first line, bevacizumab or aflibercept may be used second line^81^ ^,^ ^94^.
e) after progression to fluoropyrimidine, oxaliplatin, irinotecan, bevacizumab and/or aflibercept and cetuximab or panitumumab (if wild-type KRAS and NRAS), regorafenib is indicated, if available^97^.

#### Response evaluation

Response evaluation is recommended with reproducible imaging methods (CT, MRI or PET-CT). CEA is used in conjunction with imaging and should not be used as the sole criteria for evaluating response^98^ ^,^ ^99^.

#### Chemoembolization

Chemoembolization is a valid option in cases of illness exclusively or predominantly hepatic. In these cases, the use of microspheres of irinotecan (DEBIRI) is the treatment of choice. There is no evidence that defines which treatment line is best indicated^100^.

### RECOMMENDATIONS

• Palliative treatment is defined as cases that would not have an R0 resection, even after conversion therapy, in order to increase overall survival and/or progression-free survival and the control of symptoms, with minimal side effects.

Agreement: 94%

• Response evaluation is recommended with reproducible imaging methods (CT, MRI or PET-CT), CEA should be used in conjunction with these tests and not as the sole criterion for evaluating response.

Agreement: 96%

• The initial chemotherapy protocol can include fluoropyrimidine in monotherapy, fluoropyrimidine associated with oxaliplatin or irinotecan or a combination of the three classes of drugs. After best response, it can be maintained in full, scaled for monotherapy (maintenance) or interrupted (chemotherapy-free interval), according to clinical conditions and response to treatment.

Agreement: 98%

• For biological agents (aflibercept, bevacizumab, cetuximab and panitumumab) there is no definitive evidence that sequence or combination chemotherapy is superior. Cetuximab and panitumumab should be used in wild-type RAS patients only and regorafenib after exposure to all other classes of drugs.

Agreement: 96%

• When chemoembolization is chosen, DEBIRI is the agent of choice in exclusive or predominant liver disease.

Agreement: 77%
